# Mechanisms of Pathogenic Tau and Aβ Protein Spreading in Alzheimer’s Disease

**DOI:** 10.3389/fnagi.2020.00265

**Published:** 2020-08-27

**Authors:** Paolo d‘Errico, Melanie Meyer-Luehmann

**Affiliations:** ^1^Department of Neurology, Medical Center, University of Freiburg, Freiburg, Germany; ^2^Faculty of Medicine, University of Freiburg, Freiburg, Germany; ^3^Center for Basics in NeuroModulation (NeuroModulBasics), Faculty of Medicine, University of Freiburg, Freiburg, Germany

**Keywords:** Alzheimer’s disease, amyloid-β, tau, propagation, spreading

## Abstract

Alzheimer’s disease (AD) is pathologically defined by extracellular accumulation of amyloid-β (Aβ) peptides generated by the cleavage of amyloid precursor protein (APP), strings of hyperphosphorylated Tau proteins accumulating inside neurons known as neurofibrillary tangles (NFTs) and neuronal loss. The association between the two hallmarks and cognitive decline has been known since the beginning of the 20th century when the first description of the disease was carried out by Alois Alzheimer. Today, more than 40 million people worldwide are affected by AD that represents the most common cause of dementia and there is still no effective treatment available to cure the disease. In general, the aggregation of Aβ is considered an essential trigger in AD pathogenesis that gives rise to NFTs, neuronal dysfunction and dementia. During the process leading to AD, tau and Aβ first misfold and form aggregates in one brain region, from where they spread to interconnected areas of the brain thereby inducing its gradual morphological and functional deterioration. In this mini-review article, we present an overview of the current literature on the spreading mechanisms of Aβ and tau pathology in AD since a more profound understanding is necessary to design therapeutic approaches aimed at preventing or halting disease progression.

## The Spread of Tau

The tau protein is a phosphoprotein that is codified by alternative splicing of the microtubule-associate protein tau (MAPT) gene (Goedert et al., 1988, 1989; Andreadis et al., 1992; Andreadis, 2005; Pittman et al., 2006) and is enriched in axons of mature neurons where it regulates microtubule stability to ensure proper cytoskeletal organization and trafficking (Aamodt and Williams, 1984; Aronov et al., 2001; González-Billault et al., 2002; Zhang et al., 2004; Bertrand et al., 2013). Of all different post-translational modifications that tau can undergo, the phosphorylation is of particular interest because of its involvement in a group of neurodegenerative disorders known as tauopathies (Goedert and Spillantini, 2011; Arendt et al., 2016), including Alzheimer’s disease (AD). Indeed, whereas phosphorylation is fundamental for tau function under physiological conditions, the affinity of tau for tubulin decreases under pathological conditions, and the protein starts to accumulate in the cytosol of the somatodendritic compartments where insoluble structures are built. Those neurofibrillary tangles (NFTs) disturb the microtubule network and alter the normal axoplasmatic flow, which in turn compromises the functions and viability of neurons (Brion et al., 1985; Wood and Zinsmeister, 1989; Gastard et al., 2003). In AD, the development of NFTs evolves in the brain with a predictable and hierarchical distribution pattern that starts from layer II of the entorhinal cortex, spreads through the limbic and associations areas to finally reach the hippocampus and neocortex (Braak and Braak, 1991). Pathological tau can distribute from one cell to another thereby propagating the pathology from affected to interconnected healthy areas of the brain, implicating similar mechanisms than in prion diseases (Liu et al., 2012; Jucker and Walker, 2013). A large number of pieces of evidence support a prion-like model for tau spreading, consisting of abnormal proteins with the capacity to convert normal proteins into a pathological form. The inoculation of brain extracts from mice or humans with tauopathy into the brain of wild-type animals induced tau pathology in recipient animals and its propagation from the site of injection along neuronal connections (Clavaguera et al., 2009, 2013; Lasagna-Reeves et al., 2012; Ahmed et al., 2014; Guo et al., 2016; Gibbons et al., 2017; Narasimhan et al., 2017; Smolek et al., 2019a,b). Similar results were obtained when synthetic tau fibrils were injected into young mice overexpressing mutant human tau (P301S) which resulted as well in the formation of NFT-like inclusions that propagated from the injected sites to connected brain regions in a time-dependent manner (Iba et al., 2013). Experiments of human tau viral induction in cortical neurons in young *vs* old mice showed age- and brain region-dependent misfolding and spreading of tau (Wegmann et al., 2019). Furthermore, *in vitro* studies showed, that extracellular aggregates of tau can be internalized by naïve cells promoting fibrillization of intracellular tau that can be transferred between co-cultured cells (Frost et al., 2009; Guo and Lee, 2011, 2013) also *via* synaptic contacts between neurons that facilitate pathology propagation (Calafate et al., 2015). Tau propagation was extensively studied and different mechanisms involved in trans-cellular diffusion were described. The prion-like propagation implies an active and regulated passage of tau in the extracellular space (secretion) and a mechanism of tau uptake by an adjacent recipient cell, although the passive release of tau from dying cells cannot be excluded as an alternative scenario.

Tau filaments can exist in the brain as a variety of distinct conformational strains associated with various tauopathy phenotypes and different rates of network propagation (Sanders et al., 2014; Guo et al., 2016; Kaufman et al., 2016; He et al., 2020). Although the distribution of tau with different conformations can be suggested as cause also of AD heterogeneity, high-resolution analysis of tau structures by using cryo-electron microscopy recently revealed no significant variation in tau filament structures within and between the brains of individuals with AD (Fitzpatrick et al., 2017; Falcon et al., 2018).

**Figure 1:**
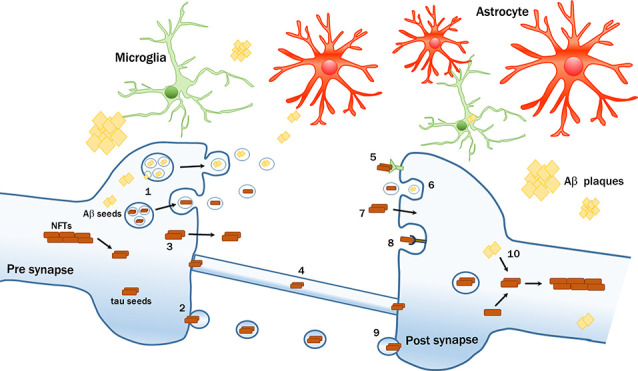
Intercellular transmission of pathological amyloid-β (Aβ) and tau proteins. Seeds of pathological proteins can be released at the presynaptic level: (1) in exosomes after the fusion of Multivesicular bodies (MVBs) with plasma membrane (PM); (2) in larger vesicles called ectosomes; (3) as naked protein freely crossing the PM; or (4) can be transferred *via* tunneling nanotubes. Mechanisms of up-take by recipient neurons include; (5) receptors-mediated endocytosis; (6) bulk-endocytosis; (7) fluid-phase translocation; (8) macropinocytosis mediated by heparan sulfate proteoglycans (HSPGs); and (9) fusion of large tau-containing large vescicles with PM. (10) Intraneuronal Aβ seeds can trigger or enhance the formation of tau pathological aggregates. The transmission process can be modulated by multiple factors, including glial cells.

Tau can be actively secreted by neurons following three main routes (Figure 1):

First, at the presynaptic level, tau can be packed into microvesicles and further released by a process (Simón et al., 2012; Fontaine et al., 2016), that is modulated by neuronal electrical activity (Lachenal et al., 2011; Pooler et al., 2013; Yamada et al., 2014; Sokolow et al., 2015; Wang et al., 2017). Following this route, phosphorylated tau is internalized by cytoplasmic exosomes, so-called intraluminal vesicles formed in multivesicular bodies (MVBs) that are finally released into the extracellular space after MVBs fusion with the plasma membrane (PM; Saman et al., 2012). Alternatively, tau can also be internalized in ectosomes, larger vesicles (100–500 nm in diameter) that are formed by evaginations of the PM incorporating tau (Dujardin et al., 2014). These routes are unconventional secretion pathways since they do not involve signal peptides and exclude the endoplasmic reticulum (ER)-Golgi system. Extracellular vesicles containing phospho-tau were found in the brains of transgenic mice (Baker et al., 2016; Polanco et al., 2016) and in peripheral fluids such as blood or CSF of AD patients (Saman et al., 2012; Fiandaca et al., 2015; Winston et al., 2016).

Second, the majority of tau is found extracellularly as a membrane-free form. Soluble hyperphosphorylated tau can translocate directly across the PM (Plouffe et al., 2012; Pooler et al., 2012) upon interaction with phosphatidylinositol 4,5 phosphate PI(4,5)P_2_ cholesterol and sphingolipids. The penetration and release are facilitated by the binding with heparan sulfate proteoglycans (HSPG) on the cell surface (Katsinelos et al., 2018; Mari et al., 2018; Merezhko et al., 2018). Other studies suggested that tau is secreted *via* the fusion of vesicles from ER or Golgi with the PM (Ponnambalam and Baldwin, 2003). Tau was also shown to form pore-like structures in the PM that operate like channels for the passage of pathogenic proteins, a feature that can be regulated either by pathological mutations, by tau oligomerization (Lasagna-Reeves et al., 2014; Patel et al., 2015; Merezhko et al., 2018), or by specific sequences of human tau that act as binding motifs to facilitate the secretion of pathological tau (Sayas et al., 2019).

Third, another mechanism that has also been proposed involves the passage through tunneling nanotubes, filamentous actin-containing channels that connect adjacent cells, and transport proteins intercellularly (Abounit et al., 2016; Tardivel et al., 2016).

Once released, tau can be internalized by recipient cells (Figure 1). Intracranial or peripheral administration of pathological tau (Clavaguera et al., 2014; Mudher et al., 2017) together with *in vitro* experiments have shown that tau is mainly internalized by active endocytic processes (Frost et al., 2009; Wu et al., 2013). In particular, three kinds of endocytosis were described:

Bulk-endocytosis represents the first one, where a large portion of presynaptic PM is internalized in a dynamin-dependent manner in form of vacuoles or endosomes from which multiple synaptic vesicles can subsequently be generated (Takei et al., 1996; Wu et al., 2013).

The second, actin-dependent macropinocytosis is mediated by HSPGs on the cell surface (Holmes et al., 2013; Rauch et al., 2018; Weisová et al., 2019). Recently, the silencing of the low-density lipoprotein receptor-related protein-1 (LRP1), which works in conjunction with HSPGs, was shown to block the uptake of tau oligomers *in vitro* and reduced *in vivo* the propagation of tau between neurons (Rauch et al., 2020). Other receptors can also be involved in the uptake of pathogenic tau, such as the extracellular portion of amyloid precursor protein (APP; Takahashi et al., 2015) and muscarinic receptors (Morozova et al., 2019). Recently, the cellular prion protein was also shown to act as a receptor that facilitates the uptake of tau aggregates by cultured cells (De Cecco et al., 2020).

Finally, clathrin-mediated endocytosis was also proposed as mechanism (Evans et al., 2018), but is still under debate because the use of specific clathrin inhibitors or its silencing resulted in continued tau aggregate uptake (Calafate et al., 2016).

In general, the different mechanisms of secretion and internalization depend largely on the cell types, the size, and the different tau species involved (Dujardin et al., 2018; Evans et al., 2018). Once tau is internalized, it can escape the endosomal vesicles inducing their rupture (Calafate et al., 2016; Flavin et al., 2017) and accumulates in the cytoplasm where it becomes a potential template for the misfolding of tau (Clavaguera et al., 2009; Guo et al., 2016; Figure 1). Although the biochemical mechanisms driving the conversion of normal tau into the pathological form are still unclear, different models of tau seeding were proposed (Congdon et al., 2008; Mirbaha et al., 2018).

Glial cells such as astrocytes (Martini-Stoica et al., 2018; Perea et al., 2019), oligodendrocytes (Narasimhan et al., 2017) and in particular microglia were implicated in tau spreading (Asai et al., 2015; Maphis et al., 2015; Hopp et al., 2018). Recently, a study demonstrated that microglia isolated from AD cases and mouse models of tauopathy contain tau seeds that can be released into the medium (Hopp et al., 2018), proposing that microglia can uptake tau but not to completely digest it, thus representing a possible source for tau spreading. The ability of microglia to engulf tau aggregates was already documented by different *in vitro* and *in vivo* studies (Luo et al., 2015; Bolós et al., 2016, 2017). Moreover, microglia depletion was shown to prevent tau propagation. Microglia phagocytosed and released tau-containing exosomes whereas inhibiting exosome synthesis significantly reduced tau propagation *in vitro* and *in vivo* (Asai et al., 2015). Finally, increased microglial activation has been reported not only to accelerate tau pathology and behavioral abnormalities in the human Tau mouse model of tauopathy (Bhaskar et al., 2010; Bemiller et al., 2017; Ising et al., 2019), but also its propagation in the brain (Maphis et al., 2015). Together, these studies highlight the involvement of microglia in spreading tau pathology.

## The Spread of Aβ

Aβ is a proteolytic product of APP, that is highly expressed in neurons and physiologically involved in many functions such as regulation of neurite outgrowth and axonal guidance, regulation of synaptic functions and plasticity, involvement in early nervous system development and in neuroprotection (Van den Heuvel et al., 1999; Leyssen et al., 2005; Priller et al., 2006; Young-Pearse et al., 2008; Mueller et al., 2018). APP can be processed in two different ways: in the non-amyloidogenic pathway, APP is cleaved first by α- followed by γ-secretase that cuts the protein within the Aβ domain. In the amyloidogenic pathway, APP is consecutively cut by β- and γ-secretase to be finally released extracellularly as Aβ fragments of different lengths, but mainly consist of 40 (Aβ1–40) or 42 (Aβ1–42) amino acids (Haass et al., 2012). Once released, monomeric Aβ can aggregate into different assemblies giving origin to oligomers, protofibrils, and amyloid fibrils that are insoluble and can further aggregate into amyloid plaques, while monomeric and oligomeric forms of Aβ are soluble. These different states of Aβ coexist in the AD brain making it difficult to dissect the most relevant and toxic forms concerning pathogenesis. Albeit *in vivo* studies demonstrated that Aβ plaques lead to neuronal loss, neuronal dystrophy and alters their normal neuritic functionality (Meyer-Luehmann et al., 2008, 2009; Shah et al., 2010), different studies have identified soluble oligomeric Aβ species as the toxic drivers responsible for synaptic dysfunction, in particular in the early stage of the disease (Lambert et al., 1998; Shankar et al., 2008; Koffie et al., 2009; Forloni et al., 2016). The fact that the incidence of senile plaques increases with age even in healthy subjects and that the number of plaques often does not correlate with neuronal loss and cognitive decline (Katzman, 1988; Villemagne and Rowe, 2011) nourishes the hypothesis that compact plaques may sequester toxic Aβ oligomers until they reach a saturation point (Esparza et al., 2013; Selkoe and Hardy, 2016). Although the ultimate proof for a causal relationship between fibrillar aggregates and neurodegenerative diseases has not been delivered yet, the “amyloid cascade hypothesis” is still the most prevalent theory (Hardy and Higgins, 1992; Hardy and Selkoe, 2002) with the constrain that Aβ alone is most likely not able to cause the entire damage found in AD patient brains (Ricciarelli and Fedele, 2017). In addition to their different aggregation state, Aβ was detected in the brain of AD patients in distinct misfolded strains with specific propagation properties (Qiang et al., 2017; Condello and Stöehr, 2018), suggesting that also these structural variations may modulate the disease phenotype.

Unlike tau that spreads in a highly predictive pattern as anticipated by computational systems (Fornari et al., 2019), Aβ deposition does not always follow a stereotypic spatio-temporal pattern of progression. Nevertheless, amyloid plaques in general first appear in the neocortex from where they spread into the allocortex and the subcortical regions (Thal et al., 2002; Serrano-Pozo et al., 2011; Grothe et al., 2017). As has been described above for tau, several studies on intracerebral injections of Aβ-rich brain extracts either from AD mice or patients propose that Aβ aggregation can be initiated by prion-like seeding (Kane et al., 2000; Meyer-Luehmann et al., 2006; Eisele et al., 2009; Jucker and Walker, 2013; Ziegler-Waldkirch et al., 2018; Friesen and Meyer-Luehmann, 2019; Katzmarski et al., 2020). These misfolded protein assemblies act as seeds of aggregation to accelerate the polymerization processes of normal proteins (Harper and Lansbury, 1997) that can expand from the injection site to distant regions as well as the contro-lateral side of the brain, thus suggesting a possible spread of seeded pathology *via* neuronal transport along axonally interconnected brain regions (Walker et al., 2002; Rönnbäck et al., 2012; Domert et al., 2014; Ye et al., 2015).

Aβ peptides are collected in intraluminal vesicles within MVBs and, upon fusion with the PM, the intraluminal vesicles are released into the extracellular space as exosomes (Rajendran et al., 2006; Sharples et al., 2008; Hu et al., 2009; Figure 1). Furthermore, a recent study reported that Aβ-rich exosomes isolated from AD patients can act as vehicles for cell-to-cell transfer of such toxic species in recipient cultured neurons (Sinha et al., 2018). However, even though the cell-to-cell passage of Aβ represents a plausible hypothesis, substantiated by the fact that the protein is found inside neurons (Wertkin et al., 1993; Turner et al., 1996; Gouras et al., 2000; LaFerla et al., 2007), there is no conclusive evidence for active transport of Aβ along neurons. Transplantations of WT neurons into brains of pre-depositing AD mice revealed that Aβ from the transgenic host tissue can enter and deposit within WT grafts (Meyer-Luehmann et al., 2003; Bachhuber et al., 2015; Espuny-Camacho et al., 2017), thus suggesting a possible passive extracellular diffusion of Aβ from the outside to the inside of the grafts. Although over the last few years many different groups have described glial cells as an alternative source of Aβ (Siman et al., 1989; Joshi et al., 2014) or their involvement in the formation of amyloid plaque deposition (Wisniewski et al., 1990; Venegas et al., 2017; Spangenberg et al., 2019), there are to date no studies that implicate a direct involvement of glial cells in Aβ trafficking across different areas of the brain. Since Aβ40 and Aβ42 circulate in body fluids such as plasma and CSF (Mehta et al., 2000), another potential route of Aβ diffusion from the periphery to the brain may be constituted by the vascular system. Indeed, intraperitoneal or intravenous administration of Aβ-rich extracts in pre-depositing APP23 mice promoted cerebral amyloid angiopathy (CAA; Eisele et al., 2010; Burwinkel et al., 2018) pointing again to a vascular component of circulating immune cell involvement in the spread of Aβ seeds (Cintron et al., 2015).

## Protein Cross-Seeding

It is well known that different neuropathological lesions such as Aβ, NFTs, or Lewy bodies can co-exist in the brains of AD patients (Braak and Braak, 1997; Hamilton, 2000), predicting cross protein interactions. Indeed, several studies have shown that the interaction between Aβ and tau can exaggerate AD pathology (Ribé et al., 2005; Bennett et al., 2017; He et al., 2018; Vergara et al., 2019) and that amyloid deposition, preceding the NFT formation, can actively influence tau spreading to neocortical regions (Braak and Braak, 1997; Hardy and Selkoe, 2002; Jacobs et al., 2018; Vogel et al., 2020). Furthermore, oligomeric forms of Aβ were found to be abundant in synapses of AD patients early in the disease before the appearance of phospo-tau at later stages, suggesting that soluble Aβ oligomers in synaptic terminals are associated with dementia onset and may initiate a cascade that drives phosphorylated tau accumulation and its synaptic spread (Bilousova et al., 2016).

Nevertheless, the finding that Aβ and tau deposition starts in different brain areas and follows distinct temporal sequences, argues against the idea that tau pathology may be driven exclusively by the presence of amyloid and rather speaks for an Aβ independent pathway (Raj et al., 2015; Jack et al., 2019; van der Kant et al., 2020). Tau aggregation assays with tau isolated from patients containing both lesions showed an enhanced ability to induce tau aggregates when compared to tau isolated from human cases without plaques (Bennett et al., 2017). Similar results have been obtained in double-transgenic mice overexpressing both, mutated APP and tau (Lewis et al., 2001; Hurtado et al., 2010). Furthermore concurrent cortical amyloid deposition in double transgenic mice strongly accelerated interneuronal transfer of tau and boosted its spreading to distal brain regions with an increase in neuronal loss (Hurtado et al., 2010; Pooler et al., 2015).

Intracerebral infusion of Aβ-rich extracts into tau-transgenic mice resulted in significantly more NFT formation compared to tau-rich or WT extracts (Götz et al., 2001; Bolmont et al., 2007; Vasconcelos et al., 2016) indicating that the presence of Aβ triggers the formation of tau pathology and proposing synergistic toxicity on the neuronal network. Inoculation of human AD-tau extracts into the brains of APP transgenic mice that normally do not form NFTs resulted in rapid fibrillization of endogenous tau (Bennett et al., 2017; He et al., 2018). Moreover, ipsi- and contralateral tau propagation was enhanced in tau-injected 5xFAD mice compared to tau-injected WT mice (Vergara et al., 2019).

Transgenic APPPS1+Tau mice, that express WT human tau within a mouse tau-deficient background showed that Aβ and tau can synergistically cooperate to cause a hyperactivity behavioral phenotype and resulted in a downregulation of genes involved in synaptic function (Pickett et al., 2019). Treatment strategies that aim at reducing tau levels in mice that co-express Aβ and human tau prevented neuronal loss (DeVos et al., 2018) as well as excitotoxin-induced neuronal dysfunctions (Roberson et al., 2007) and ameliorated the behavioral and gene expression phenotypes (Pickett et al., 2019). Together, these results demonstrate the therapeutic benefit of tau reduction with a positive impact on the Aβ-induced cytotoxic effects.

Despite all these observational evidence, the mechanism of Aβ and tau interplay remains largely unknown. Further studies are necessary to unravel whether it is a direct interaction between the two pathogenic proteins or instead mediated by other factors.

## Conclusions and Future Prospective

Two of the most remarkable features of AD are: (i) the stereotypic pattern of Aβ and tangle spreading through interconnected areas of the brain (Braak and Braak, 1991) that is closely related to cognitive decline years before the onset of clinical symptoms; and (ii) the ability of pathogenic misfolded Aβ and tau to serve as templates to convert their innocuous counterparts into toxic forms in a prion-like manner (Clavaguera et al., 2009; Jucker and Walker, 2011, 2013). These two aspects, together with the fact that the two hallmarks often coexist in the brain of AD patients and amplify each other’s toxic effects downstream (Ittner and Götz, 2011), make the development of an effective therapy challenging. Currently, clinical trials targeting Aβ have reported limited success, implying the notion that the timing of intervention is too late and that directing to only one target might be not sufficient to halt the spreading of both pathogenic proteins and to avoid their synergistic impact on neuronal networks. Furthermore, abundant pieces of evidence have recently highlighted the role that different strains of tau and Aβ may play in modulating the clinical picture of the disease, turning away the possibility to develop therapies against different AD subtypes. For this purpose, a better understanding of the conformational heterogeneity of tau and Aβ is necessary to better design the best intervention methods.

Although in the last years many molecular and cellular mechanisms involved in the formation, aggregation, deposition, and propagation of Aβ and tau were uncovered, many questions remain still open. Which mechanisms guide Aβ diffusion? What is the exact sequence of events leading to clinical symptoms? Which role might other cellular systems or molecular pathways play in promoting the pathological bond of Aβ and tau? These are only some of the questions that need to be clarified to design the best strategies to finally arrest disease progression.

## Author Contributions

Pd’E and MM-L wrote the manuscript. Pd’E and MM-L read and approved the final manuscript.

## Conflict of Interest

The authors declare that the research was conducted in the absence of any commercial or financial relationships that could be construed as a potential conflict of interest.
